# Psychodynamic therapy of depression

**DOI:** 10.1177/00048674211031481

**Published:** 2021-07-09

**Authors:** Falk Leichsenring, Patrick Luyten, Allan Abbass, Christiane Steinert

**Affiliations:** 1 Department of Psychosomatics and Psychotherapy, Justus Liebig University Giessen, Giessen, Germany; 2 Department of Psychosomatics and Psychotherapy, University of Rostock, Rostock, Germany; 3 Faculty of Psychology and Educational Sciences, University of Leuven, Leuven, Belgium; 4 Research Department of Clinical, Educational and Health Psychology, University College London, London, UK; 5 Department of Psychiatry, Dalhousie University, Halifax, NC, Canada; 6 Centre for Emotions and Health, Dalhousie University, Halifax, NC, Canada; 7 International Psychoanalytic University (IPU), Berlin, Germany

In this journal, Malhi et al. (2021) present the 2020 Royal Australian and New Zealand College of Psychiatrists clinical practice guidelines for mood disorders. While we applaud their efforts to develop a comprehensive treatment guideline, we call attention to several factual errors leading to erroneous conclusions and recommendations with regard to the treatment of mood disorders.

These errors refer to (1) the evidence for psychodynamic therapy in complex presentations, (2) the evidence for long-term psychodynamic therapy, (3) the stability of treatment effects, (4) the response rates achieved by psychodynamic therapy in depression and (5) the role of regression and insight in psychodynamic therapy.

We agree with Malhi et al. (2021) that short-term psychodynamic psychotherapy has proved to be efficacious in depression. However, referring to complex presentations of depression, Malhi et al. (2021) argue that for psychodynamic therapies ‘*… there are no RCTs … to suggest that they may be of some help*’ (p. 96). This statement is in clear contradiction to the evidence cited by the authors themselves some lines earlier when referring to the meta-analysis by Cristea et al. (2017). In fact, this very meta-analysis found psychodynamic therapy and dialectical behavior therapy (DBT) but not cognitive-behavioral therapy (CBT) to be superior to controls, with the descriptively largest between-group effect size for psychodynamic therapy (CBT: *g* = 0.24, DBT: *g* = 0.34, psychodynamic therapy: *g* = 0.41). Across treatments, significant improvements were found for depression, anxiety and general psychopathology (Cristea et al., 2017). Thus, the authors’ statement cited above (p. 96) is incorrect.

This also applies to a statement by Malhi et al. (2021) claiming that ‘*… there is no evidence to support … long-term psychodynamic therapy*’ (p. 44) since there is evidence showing that long-term psychodynamic therapy is effective in borderline personality disorder (BPD, Cristea et al., 2017) and in other complex presentations of depression (see Online Supplement #1).

Citing Cristea et al. (2017), Malhi et al. (2021: 96) claim that effects of psychotherapy on BPD are unlikely to be sustained at follow-up. However, this is neither true for effects of psychodynamic therapy of BPD nor for psychodynamic therapy of other complex presentations of depression, as their effects have proved to be stable (see Online Supplement #2).

For psychodynamic therapy, Malhi et al. (2021) emphasize ‘*… that not all depressive presentations benefit from this therapeutic approach*’ (p. 42). We agree, however, this is true for other approaches as well. For CBT, rates for remission and response were found to be 49% and 53%, with no differences to other forms of psychotherapy (Cuijpers et al., 2014). For selective serotonin reuptake inhibitors, response rates of 51% vs 39% for placebo were reported (see Online Supplement #3).

Furthermore, again only for psychodynamic therapy ‘*robust replications*’ are emphasized as necessary (Malhi et al., 2021: 42), implying a caveat for one form of therapy only. Independent and unbiased replications are definitely necessary, but for all approaches (see Online Supplement #4).

Malhi et al. (2021) argue that ‘*Psychodynamic therapies promote regression, which can be distressing for some patients, and even generate transitory deterioration in mental state*’ (p. 43). However, neither treatment manuals of short-term psychodynamic therapy for depression nor manuals for the long-term treatment of complex presentations of depression (e.g. with comorbid BPD) promote regression; by contrast, regression is explicitly restricted in these manuals (e.g. Leichsenring and Steinert, 2018). Even more importantly, both efficacy and effectiveness studies of psychodynamic therapy for depression show consistent linear or quadratic *decreases* of depressive symptoms, even in studies reporting session-by-session assessment of depressive symptoms (see Online Supplement #5). Regression is only promoted in classical psychoanalysis for patients who are able to tolerate it.

Another statement by Malhi et al. (2021) on psychodynamic therapy not supported by any evidence is that ‘*… the development of insight is very important in this form of therapy, and some patients struggle to apply this new knowledge*’ (p. 43). Patients may struggle to do so just as patients in CBT may struggle with exposure techniques, or patients on antidepressants with side effects, but process-outcome research has shown that gaining insight is related to outcome in psychodynamic therapy and in other therapies too (see Online Supplement #6). Of note, in psychodynamic therapy, insight does not only include cognitive processes but also emotional experiencing and understanding (see Online Supplement #7).

As another issue, Malhi et al. (2021: 90) state that there is level I evidence for fluoxetine in the treatment of young people with depression, citing a network meta-analysis by Zhou et al. (2020). This meta-analysis, however, suffers from serious methodological shortcomings (see Online Supplement #8).

Some references given by Malhi et al. (2021) need correction (see Online Supplement #9).

In sum, the incorrect statements by Malhi et al. (2021) result in an inappropriately negative view of psychodynamic therapy for depression. A more balanced evaluation of the relevant evidence is needed.

## Supplemental Material

sj-docx-1-anp-10.1177_00048674211031481 – Supplemental material for Psychodynamic therapy of depression Click here for additional data file. Supplemental material, sj-docx-1-anp-10.1177_00048674211031481 for Psychodynamic therapy of depression by Falk Leichsenring, Patrick Luyten, Allan Abbass and Christiane Steinert in Australian & New Zealand Journal of Psychiatry
