# Supplementary material for: Psychodynamic therapy of depression
Source: Aust N Z J Psychiatry. 2021 Jul 9;55(12):1202–3. doi: 10.1177/00048674211031481 (PMC8647477; doi:10.1177/00048674211031481)
Supplement: sj-docx-1-anp-10.1177_00048674211031481 – Supplemental material for Psychodynamic therapy of depression [file sj-docx-1-anp-10.1177_00048674211031481.docx]

**Supplementary References**

#1

Knekt, P., Lindfors, O., Harkanen, T., Valikoski, M., Virtala, E., Laaksonen, M. A., et al. (2008). Randomized trial on the effectiveness of long-and short-term psychodynamic psychotherapy and solution-focused therapy on psychiatric symptoms during a 3-year follow-up. *Psychological Medicine, 38*, 689-703.

Huber, D., Zimmermann, J., Henrich, G., & Klug, G. (2012). Comparison of cognitive-behavior therapy with psychoanalytic and psychodynamic therapy for depressed patients - a three-year follow-up study *Zeitschrift für Psychsomatische Medizin und Psychotherapie, 58,*  299-316.

Leuzinger-Bohleber, M., Hautzinger, M., Fiedler, G., Keller, W., Bahrke, U., Kallenbach, L., et al. (2019). Outcome of Psychoanalytic and Cognitive-Behavioural Long-Term Therapy with Chronically Depressed Patients: A Controlled Trial with Preferential and Randomized Allocation. *Can J Psychiatry, 64*(1), 47-58. doi: 10.1177/0706743718780340

Fonagy, P., Rost, F., Carlyle, J. A., McPherson, S., Thomas, R., Pasco Fearon, R. M., et al. (2015). Pragmatic randomized controlled trial of long-term psychoanalytic psychotherapy for treatment-resistant depression: the Tavistock Adult Depression Study (TADS). *World Psychiatry, 14*(3), 312-321. doi: 10.1002/wps.20267

#2

Bateman, A. & Fonagy, P. (2008). 8-year follow-up of patients treated for borderline personality disorder: mentalization-based treatment versus treatment as usual. *Am J Psychiatry* 165, 631-8.

Smits, M. L., Feenstra, D. J., Bales, D. L., Blankers, M., Dekker, J. J. M., Lucas, Z., Kamphuis, J. H., Busschbach, J. J. V., Verheul, R. & Luyten, P. (2020). Day hospital versus intensive outpatient mentalization-based treatment: 3-year follow-up of patients treated for borderline personality disorder in a multicentre randomized clinical trial. *Psychol Med*, 1-11.

Fonagy, P., Rost, F., Carlyle, J. A., McPherson, S., Thomas, R., Pasco Fearon, R. M., et al. (2015). Pragmatic randomized controlled trial of long-term psychoanalytic psychotherapy for treatment-resistant depression: the Tavistock Adult Depression Study (TADS). *World Psychiatry, 14*(3), 312-321. doi: 10.1002/wps.20267

Town JM, Abbass A, Stride C, Bernier D. (2017) A randomised controlled trial of Intensive Short-Term Dynamic Psychotherapy for treatment resistant depression: the Halifax Depression Study. J Affect Disord 214:15-25. doi: 10.1016/j.jad.2017.02.035.

Town JM, Abbass A, Stride C, Nunes A, Bernier D, Berrigan P (2020). Efficacy and cost-effectiveness of intensive short-term dynamic psychotherapy for treatment resistant depression: 18-Month follow-up of the Halifax depression trial. J Affect Disord. 2020 273:194-202. doi: 10.1016/j.jad.2020.04.035.

#3

Barth, M., Kriston, L., Klostermann, S., Barbui, C., Cipriani, A. & Linde, K. (2016). Efficacy of selective serotonin reuptake inhibitors and adverse events: meta-regression and mediation analysis of placebo-controlled trials. *British Journal of Psychiatry* 208, 114-9.

# 4

Leichsenring, F., Abbass, A., Hilsenroth, M. J., Leweke, F., Luyten, P., Keefe, J. R., et al. (2017). Biases in research: risk factors for non-replicability in psychotherapy and pharmacotherapy research. *Psychological Medicine, 47*(6), 1000-1011. doi: 10.1017/S003329171600324X

#5

Driessen, E., Hegelmaier, L. M., Abbass, A. A., Barber, J. P., Dekker, J. J., Van, H. L.,

Jansma, E. P. & Cuijpers, P. (2015). The efficacy of short-term psychodynamic psychotherapy for depression: A meta-analysis update. *Clinical Psychology Review* 42, 1-15.

Fonagy, P., Lemma, A., Target, M., O'Keeffe, S., Constantinou, M. P., Ventura Wurman, T., Luyten, P., Allison, E., Roth, A., Cape, J. & Pilling, S. (2020). Dynamic interpersonal therapy for moderate to severe depression: a pilot randomized controlled and feasibility trial. *Psychol Med* 50, 1010-1019.

Lemma, A., Target, M. & Fonagy, P. (2011). The development of a brief psychodynamic

intervention (Dynamic Interpersonal Therapy) and its application to depression: A pilot study. *Psychiatry: Interpersonal and Biological Processes* 74, 41-48.

#6

Crits-Christoph, P., Connolly Gibbons, M. B., & Mukherjee, D. (2013). Psychotherapy processs outcome research. In M. J. Lambert (Ed.), *Bergin and Garfield´s handbook of psychotherapy and behavior change* (6 ed., pp. 298-340). New York: Wiley.

Jennissen, S., Huber, J., Ehrenthal, J. C., Schauenburg, H. & Dinger, U. (2018). Association Between Insight and Outcome of Psychotherapy: Systematic Review and Meta-Analysis. *American Journal of Psychiatry* 175, 961-969.

Høglend, P., & Hagtvet, K. (2019). Change mechanisms in psychotherapy: Both improved insight and improved affective awareness are necessary. Journal of Consulting and Clinical Psychology 87, 332-344.

#7

Crits-Christoph, P., Crits-Christoph, K., Wolf-Palacio, D., Fichter, M. & Rudick, D. (1995).

Brief supportive-expressive dynamic psychotherapy for generalized anxiety disorder. In *Dynamic therapies for psychiatric disorders: Axis I* (ed. J. P. Barber and P. Crits-Christoph), pp. 43-83. Basic Books: New York.

Caldiroli, A., Capuzzi, E., Riva, I., Russo, S., Clerici, M., Roustayan, C., et al. (2020). Efficacy of intensive short-term dynamic psychotherapy in mood disorders: A critical review. *J Affect Disord, 273*, 375-379. doi: 10.1016/j.jad.2020.04.002

Blagys, M. D., , & Hilsenroth, M. J. (2000). Distinctive features of short-term psychodynamic-interpersonal psychotherapy: a review of the comparative psychotherapy process literature. *Clinical Psychology Science & Practice* 7, 167-188.

#8

Leichsenring, F., Luyten, P., Abbass, A., Rabung, S. & Steinert, C. (2021). Treatment of depression in children and adolescents. *Lancet Psychiatry* 8, 96-97.

#9

The references given by Malhi *et al.* (2021, p. 42, 43) for the controversy between Hofmann and Leichsenring et al. and for the evidence of psychodynamic therapy are incorrect. The correct references are given below.

Leichsenring, F., Abbass, A., Gottdiener, W., Hilsenroth, M., Keefe, J. R., Luyten, P.,

Rabung, S. & Steinert, C. (2016). Psychodynamic therapy: a well-defined concept with increasing evidence. *Evidence-Based Mental Health* 19, 64.

Leichsenring, F., Leweke, F., Klein, S., & Steinert, C. (2015). The empirical status of psychodynamic psychotherapy - an update: Bambi's alive and kicking. *Psychotherapy and Psychosomatics, 84*(3), 129-148. doi: 10.1159/000376584
